# Remarks

**Published:** 1866-12

**Authors:** S. H. Stout


### REMARKS.

The reports from which the lists of deaths at Pensacola, in former numbers of this Journal, have been taken, are duplicates of those made by Surg. A. J. Foard, Medical Director, (now residing at Columbus, Ga.,) which he has kindly permitted me to use. To avoid misapprehension among those of your readers who were not in the Confederate Army, it is proper to remark that Gen. Foard was on the staff of Gen. Braxton Bragg, during nearly all of his career. The little army at Pensacola was a complete organization in all its departments. The Medical Department, as well in the field as in the hospitals, was organized by Dr. Foard, and was, at the early period to which these reports relate, as complete, and as well disciplined, as any department of the Confederate Army at any subsequent period. During his stay at Pensacola, Dr. Foard twice (in one instance, as early as May or June 1861,) successfully performed the operation of amputation at the shoulder joint; complete recovery following in both instances.

Prior to the battle of Shiloh, Gen. Bragg's army left Pensacola, and made a junction at Corinth, with Gen. A. S. Johnson. There, by virtue of his rank, Dr. Foard succeeded Surgeon D. W. Yandell as Medical Director of Department No. 2, which extended from the Department of E. Tennessee, commanded by Gen. E. Kirby Smith, to the Mississippi River. When the army moved to Chattanooga, prior to the Kentucky campaign, which resulted in the battle of Perryville, he conceived the idea of placing the hospitals in the rear, in charge of a superintendent, who should report to headquarters through him, and be guided by his direction. For this important and responsible position the writer was chosen. In June 1863, his position was modified by orders from the War Department, by being denominated a Director of Hospitals, with directions to report direct to the Surgeon General. This modification did not practically change the relationship to a very great extent. For the necessities of the service demanded, at all times, a cordial co-operation of the Medical Director in the field, and of the Medical Director of Hospitals. At no time was Surgeon Foard subordinate to me, and while his subordinate, it was a pleasure to myself and others of the medical staff, to honor him as our chief.

Having had chief control of the Medical Department of the western army, after the battles of Shiloh, Perryville, Murfreesboro—during Joseph E. Johnston's celebrated campaign in Georgia; during Hood's commandership of the army, both in Georgia and in Tennessee, and only laid aside his office at the termination of the war—Dr. Foard has had opportunities of acquiring practical experience in regard to military surgery, discipline, hygiene, and everything that relates to the medical department of armies, which has fallen to the lot of few men in any age or country. That he performed his duties well, the almost universal acclaim of his subordinates testifies. No man in the medical staff of the army made more sacrifices, and none brought to his aid more minute knowledge of his duties. For he occupied before the war an honorable position in the army of the United States, and promptly left it in obedience to the honorable impulses of a native Georgian, when his State seceded.

After the battle of Murfreesboro, Dr. Foard left the staff of Gen. Bragg, and was succeeded by Surgeon E. A. Flewellen, now of Thomaston, Georgia, to take the position of Medical Director on the staff of Gen. Joseph E. Johnston, whose command then extended from the mountains to the Mississippi River, and included several Departments. This position was afterwards changed to that of Medical Inspector of the Departments referred to. After the battle of Missionary Ridge had been fought, Dr. Flewellen having been relieved at his own request, on account of ill health, Dr. Foard was again assigned as Medical Director of the Army of Tennessee.

To the earnest interest and the cordial support which

Medical Directors Foard and Flewellen ever manifested, the Medical Director of the Hospitals of the Army of Tennessee attributes much of his success in performing his difficult and arduous task; and if any honor or gratitude of the profession or of the soldiery is due the latter, he would not have the former forgotten or overlooked.

I can not but hope that the time will yet arrive, when circumstances surrounding us will admit of our jointly furnishing the public with such a history of those who served under us, as their directors, so cheerfully, so earnestly, so skillfully, and whose labors, under difficulties, were crowned with a success of which the medical staff of armies far better supplied, might, with much propriety, congratulate themselves.

S. H. Stout.

---

We regret the failure to receive from our colleague and contributor to the Journal, Dr. Stout, the usual monthly amount of Army Statistics, for the present number. The above statement, explanatory of the rank of, and connection between the several Medical Directors belonging to the Army of Tennessee, was received some weeks since, with a promise of the ordinary amount of Hospital Reports, for December. It has not, however, reached us before going to press, notwithstanding the present issue is delayed several days beyond the usual time of publication.

